# Rapid Preparation of Fluorescent Carbon Dots from Pine Needles for Chemical Analysis

**DOI:** 10.3390/nano12010066

**Published:** 2021-12-28

**Authors:** Hweiyan Tsai, Kaiying Chang, Wanshing Lee, C. Bor Fuh

**Affiliations:** 1 Department of Medical Applied Chemistry, Chung Shan Medical University, Taichung 402, Taiwan; annetsai@csmu.edu.tw; 2 Department of Medical Education, Chung Shan Medical University Hospital, Taichung 402, Taiwan; 3 Department of Applied Chemistry, National Chi Nan University, Puli, Nantou 545, Taiwan; s108324501@mail1.ncnu.edu.tw (K.C.); s108324902@mail1.ncnu.edu.tw (W.L.)

**Keywords:** carbon dots, pine needles, chemical analysis, carcinoembryonic antigen, tumor necrosis factor-alpha

## Abstract

Fluorescent carbon dots with blue, green, and red emissions were rapidly prepared from modified pine needles through microwave irradiation in a one-pot reaction. The fluorescence intensity and emission versatility for a carbon source were experimentally optimized. The reaction times were under 10 min and the reaction temperatures were lower than 220 °C. Potential applications of magnetic fluorescence-linked immunoassays of carcinoembryonic antigen (CEA) and tumor necrosis factor-alpha (TNF-α) were presented. The detection limits for CEA and TNF-α (3.1 and 2.8 pg mL^−1^, respectively) are lower than those presented in other reports, whereas the linear ranges for CEA and TNF-α (9 pg mL^−1^ to 18 ng mL^−1^ and 8.5 pg mL^−1^ to 17 ng mL^−1^, respectively) are wider than those presented in other reports. Magnetic immunoassays with fluorescent CD_s_ prepared from pine needles can enable rapid, sensitive, and selective detections for biochemical analysis.

## 1. Introduction:

In general, carbon dots (CD_s_) are carbon nanoparticles with a size of less than 10 nm. Carbon dots have optical, electrical, and chemical properties suitable for various applications [1,2,3,4,5,6,7]. Fluorescent CD_s_ are widely used in numerous chemical, biochemical, and environmental applications because of their hydrophilicity, biocompatibility, biodegradability, and stability features with respect to organic dyes and inorganic quantum dots. 

The fluorescence properties of CD_s_ depend on their synthesis procedure and the precursors used in their preparation. Most CD_s_ display blue fluorescence and some CD_s_ exhibit green fluorescence. Green carbon sources can be used to produce widely available, cost-effective, and ecologically friendly CD_s_. Green carbon precursors have gained wide popularity in scientific worlds to support sustainable environments. Thus, developing a simple, rapid, green, and economical method for synthesizing CD_s_ with various emission wavelengths for optical sensing is crucial.

Pine needles come from pinus morrisonicola (five-leaf pines) which are grown in the mountainous regions of Taiwan at elevations of 300–2300 m. Pine needle-related products are used in dietary supplements and have potential antioxidative, antitumor, and antibacterial functions [8,9,10,11]. Pine needles are green carbon sources with high potentials for use in producing fluorescent CD_s_ with various emission wavelengths. Microwave heating is a homogeneous, efficient, cost-effective, and scalable heating method with a short reaction time. Microwave-assisted synthesis on nanomaterials is eco-friendly and has become popular in scientific research [12,13]. Herein, fluorescent CD_s_ with various emission wavelengths were rapidly prepared from modified pine needles through microwave irradiation.

Biomarker detections have been used as an essential reference for the early diagnosis of cancer-related diseases and for monitoring the progress of medical treatments [14,15]. Currently, the enzyme-linked immunosorbent assay (ELISA) is the most commonly used biomarker detection approach; however, it is highly labor-intensive and time-consuming. Magnetic immunoassays with functional nanoparticles have been reported to provide higher detection speed and detection sensitivity than does ELISA [16,17,18,19]. Magnetic nanoparticles of iron oxides can provide a fast reconcentration of immunocomplexes after reaction and washing. Magnetic immunoassays with functional CD_s_ were employed for biomarker detection to demonstrate the potential applications. Herein, carcinoembryonic antigen (CEA) and tumor necrosis factor-alpha (TNF-α) were used as representative biomarkers.

## 2. Experimental Method

### 2.1. Materials

Tetraethoxysilane (TEOS), ethylenediamine (≥99%), phosphoric acid (≥85%), 3-aminopropyl triethoxysilane (≥99%), cetyltrimethylammonium bromide (CTAB, ≥98%), 1-ethyl-3-(3-dimethyl aminopropyl) carbodiimide (EDC, ≥98%), N-hydroxysuccinimide (NHS, ≥99%), CEA, and monoclonal and polyclonal anti-CEA, were purchased from Sigma-Aldrich (St. Louis, MO, USA). TNF-α and anti-TNF-α were acquired from Fitzgerald Industries International (Aton, MO, USA). Sera samples were supplied by Jackson Immunoresearch (West Grove, CA, USA), and ferrous and ferric chlorides (FeCl_2_ 4H_2_O, FeCl_3_ 6H_2_O) were obtained from J. T. Baker (Philipsburg, NJ, USA). Double distilled deionized water was from Milli-Q (Fremont, CA, USA) with a resistivity of 18.2 MΩ·cm. The microwave instrument was purchased from Milestone (ETHOS UP, Sorisole, Italy). The UV-vis spectrometer was from Agilent Scientific (HP8450, Santa Clara, CA, USA), the FTIR was from Perkin Elmer (Spectrum 2, Waltham, MA, USA), the TEM was from Joel (JEL 1400, Tokyo, Japan), and the superconducting quantum interference device (SQUID) magnetometer was from Quantum Design (San Diego, CA, USA).

### 2.2. Preparation of Functional Nanoparticles

Pine needles were collected from the university campus of National Chi Nan University at Puli, Nantou, Taiwan. They were then washed with water, air-dried, placed in an oven at 60 °C for 1.5 h, cut into small pieces, and ground into fine powder. The powder was stored in a dry box for further use.

Three types of CD_s_ (CD_b_, CD_g_, and CD_r_) were prepared through a one-pot microwave reaction by using the pine needle powder as the main carbon source. The reaction temperature and reaction time of the microwave were optimized to obtain the fluorescence wavelength and intensity. The ranges of temperature were 160 to 220 °C for CD_b_ and CD_g_ and extended down to 40 °C for CD_r_. The ranges of running time were from 5 to 20 min. CD_b_ was prepared by placing a mixture of 0.1 g of needle powder, 0.2 mL of ethylenediamine, 1 mL of phosphoric acid, and 10 mL of distilled water in a microwave at 220 °C for 10 min. Next, the mixture was centrifuged at 8000 rpm for 15 min by using a membrane tube with a molecular weight cutoff of 3000 MW. The upper part of the mixture was collected, vacuum dried, and stored for further use. CD_g_ was prepared through the same procedure as was CD_b_, except that no phosphoric acid was added to the mixture before the microwave reaction. CD_r_ was prepared by mixing 0.1 g of needle powder with 10 mL of ethanol and placing the mixture in a microwave at 50 °C for 5 min. The solution was centrifuged at 8000 rpm for 15 min by using a membrane tube with a molecular weight cutoff of 1000 MW. The upper part of the solutions was collected, vacuum dried, and stored for further use. The concentrations of fluorescent and UV-vis measurement in PBS were 1 mg mL^−1^ and 0.1 mg mL^−1^, respectively. The quantum yield (QY) was calculated based on the following equation. QY_c_ = QY_s_ × (I_c_/I_s_) × (A_s_/A_c_) × (N_c_^2^/N_s_^2^). Where QY is the quantum yield, the subscripts “c” and “s” refer to CD_s_ and standard, respectively. I is the integrated area in the emission spectrum and A is the absorbance at 320 and 410 nm wavelength. Quinine sulfate (QY_s_ = 54%, N_s_ = 1.33 in 0.1 M H_2_SO_4_ solution) and Rhodamine 6 G (QY_s_ = 95%, Ns = 1.36 in ethanol) were employed as the fluorescence standards.

Iron oxides were prepared through chemical precipitation. In brief, 0.27 g of ferric chloride and 0.10 g of ferrous chloride in 25 mL of distilled water were added with 4.5 mL of ammonium hydroxide. Iron oxide @mSiO_2_ was prepared by mixing 25 mL of a solution containing 0.15 g of CTAB, 0.05 g of iron oxide, 0.15 mL of TEOS, 2.4 mL of ethanol, and 1.8 mL of ammonium hydroxide solution (28%). Iron oxide @mSiO_2_ was subjected to amination by mixing 1 mL of 3-aminopropyl triethoxysilane and 1 ml of NH_4_OH. This mixture was coupled with CD_b_ by mixing 1 mL of CD_b_ (0.12 g mL^−1^) with 0.5 mL of 0.3 M EDC, 0.5 mL of 0.075 M NHS, and 2 mg of iron oxide @mSiO_2_ for 4 h. The coupling of various nanoparticles (3.0 × 10^15^) with biomarker antibodies (2 mL of 8.0 × 10^−8^ M) was through a reaction involving the combination of 0.023 g of NHS and 0.153 g of EDC with 10 mL of phosphate-buffered saline over 2 h.

### 2.3. Magnetic Fluorescence-Linked Immunoassay

**Figure 1:**
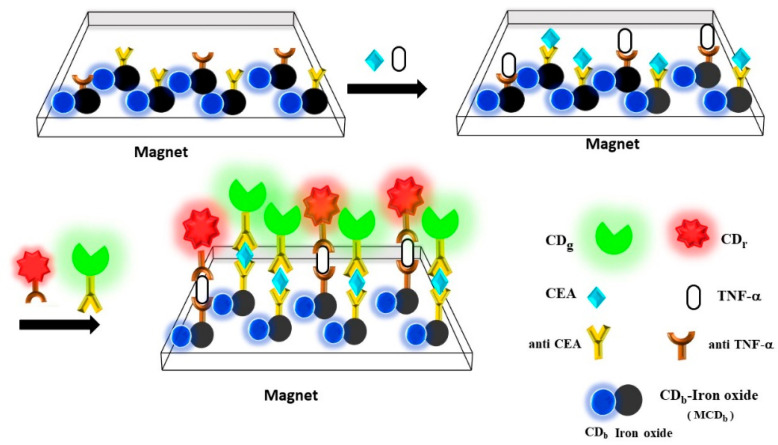
Schematic representation of the magnetic fluorescence-linked immunoassay with magnetofluorescent and fluorescent nanoparticles for the detection of CEA and TNF-α.

Figure 1 displays magnetofluorescent nanoparticles (iron oxide @mSiO_2_-CD_b_) that were labeled with anti-CEA and anti-TNF-α in a microplate well. A magnetic microplate was prepared by placing permanent magnets of rare earth (13 mm in length and 6 mm in diameter) under the bottom of a microplate for particle reconcentration after mixing and washing. The magnetic field strength was 2.5 ± 0.1 kG at the bottom well of the microplate. The volume of each reactant solution was 100 μL. Various quantities of CEA and TNF-α were allowed to react with magnetofluorescent labeled nanoparticles for immunocomplex formation. The presence of complexes containing CEA and TNF-α was confirmed using fluorescence (CD_g_ and CD_r_)-labeled anti-CEA and anti-TNF-α, respectively. Each step of reaction included mixing and reconcentration to remove unreacted species under a magnetic field. The numbers of magnetofluorescent, CD_g_@mSiO_2_, and CD_r_@SiO_2_ nanoparticles labeled with the antibody used in the sandwich reaction of magnetic fluorescence-linked immunoassays were 3.0 × 10^1^^2^, 1.5 × 10^13^, and 1.5 × 10^13^, dispersed in 100 μL of solutions, respectively.

The reference plots of CEA and TNF-α were established by adding known concentrations of CEA and TNF-α (10^−6^ M to 10^−16^ M) to the sandwich reaction and then confirming the added concentrations by using anti-CEA-labeled and anti-TNF-α-labeled CDs. The relative fluorescent intensity (F/F_o_) is the ratio of the fluorescent intensity in the sandwich complex (F) to the fluorescent intensity before the sandwich reaction (F_o_).

## 3. Results and Discussion

### 3.1. Characterization of Nanoparticles

**Scheme 1:**
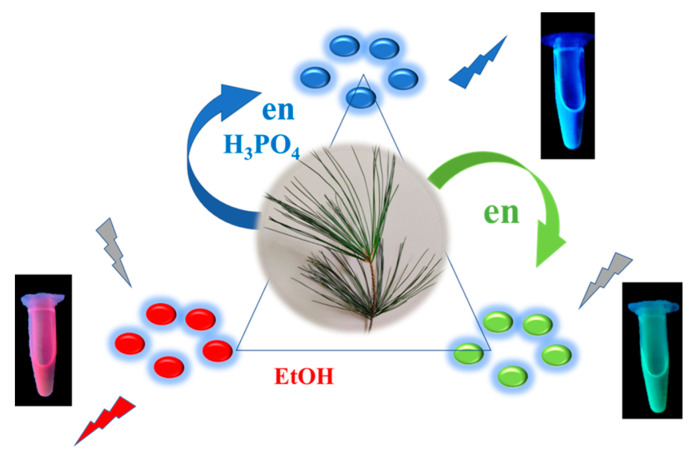
Preparation for various CD_s_.

**Figure 2:**
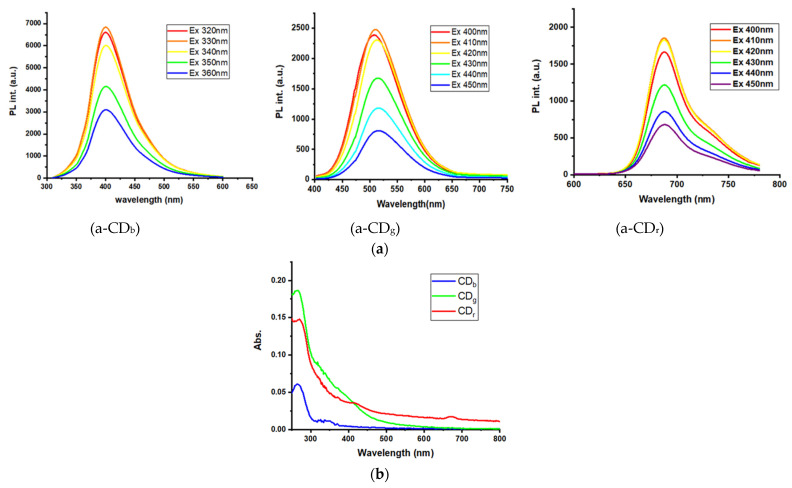
(**a**) Changes in the emission wavelengths of the maximal intensity of the three types of CD_s_ (1 mg mL^−1^ in PBS); (**b**) ultraviolet-visibleabsorption spectra of the three types of CD_s_ (0.1 mg mL^−1^ in PBS).

**Figure 3:**
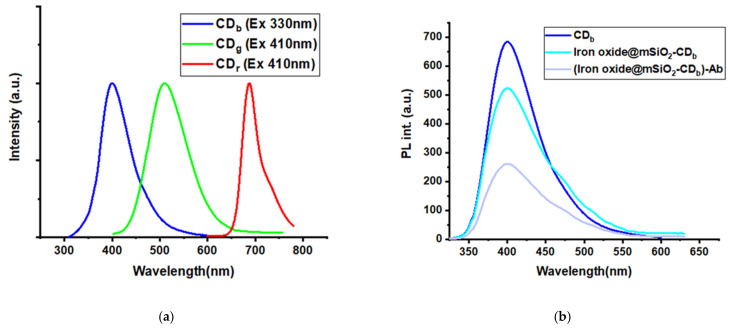

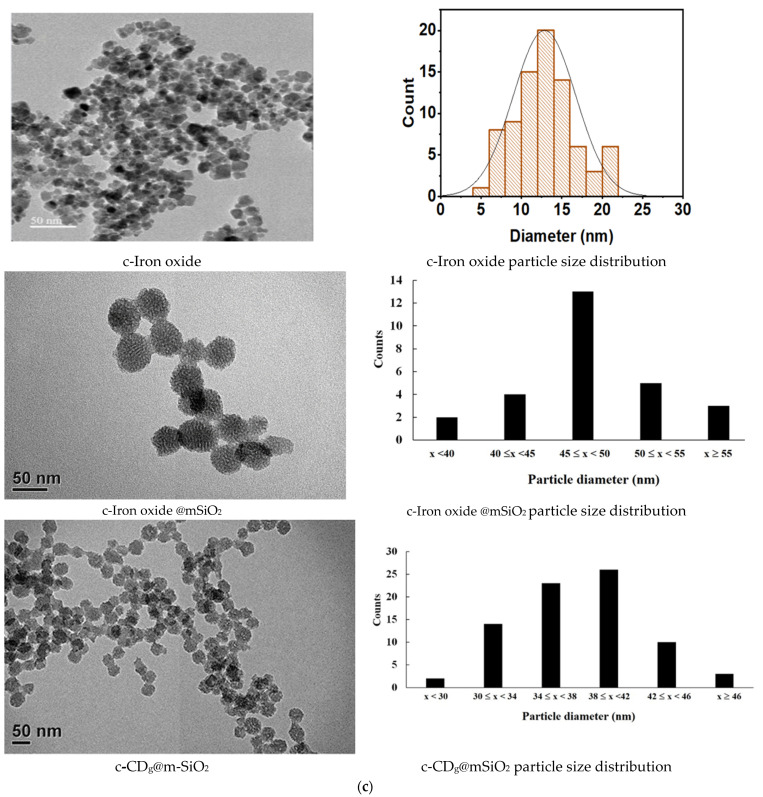
(**a**) Optimal emission spectra of the three types of CD_s_, (**b**) variations in the fluorescence intensity of CD_b_ with each modification step, and (**c**) transmission electron microcopy images of iron oxide, iron oxide @mSiO_2_, and CDg@mSiO_2_.

The preparation of CD_s_ from modified pine needles through microwave irradiation is displayed in the Scheme 1. The reaction times for all the CD_s_ were less than 10 min. The reaction temperature ranged from 180 to 220 °C and the optimal reaction temperature for CD_b_ and CD_g_ was 220 °C. The relative fluorescent intensity (FI) was used to optimize the experimental conditions. Figure S1 shows the optimization of reaction temperature for a fixed time and reaction time for a fixed temperature. The sizes of CD_b_, CD_g_, and CD_r_ nanoparticles were 3.0 ± 0.2, 4.0 ± 1.5, and 5.5 ± 1.9 nm, respectively (Figure S2). Figure S3 presents the infrared spectra of the CD_s_. The three types of CD_s_ exhibited absorption peaks at 1070, 1650, and 3400 cm^−1^ (ascribed to C–O, C=O, and O–H stretching, respectively), indicating the presence of carboxylic acid. The absorption peaks at 2981 and 2897 cm^−1^ corresponded to the C–H vibration of CD_b_ and CD_g_ nanoparticles, respectively. The emission wavelengths depended on the excitation wavelengths for the CDs. The excitation wavelength (Ex) range of CD_b_ was 320–360 nm and that of CD_g_ and CD_r_ was 400–450 nm. The emission wavelength of the maximal intensity changed marginally for each CD as shown in Figure 2a. The maximal emission intensities were optimized using appropriate excitation wavelengths. Specifically, the optimal emission wavelengths of CD_b_, CD_g_, and CD_r_ were 400, 510, and 680 nm, respectively, under excitation wavelengths of 320, 410, and 410 nm, respectively. Figure 2b shows UV-vis absorption spectra of the three types of CD_s_ in PBS. The excitation wavelengths of 320, 410, and 410 nm in fluorescence from CD_s_ were not the maximal absorption in the absorption spectra region of CD_s_ in UV-vis spectra. The excitation wavelengths were optimized by the maximal fluorescent intensity as shown in Figure 2a,b. We know that there are some intrinsic fluorescence materials in the pine needles, but we can not discern and isolate exactly from the overall emitting properties due to their complex components. Figure 3a depicts the optimal emission spectra of the three types of CD_s_. CD_b_ was used for the magnetofluorescent reaction, and CD_g_ and CD_r_ were used to detect the sandwich detection. The QY_s_ of CD_b_, CD_g_, CD_r_, CD_g_@mSiO_2_, and CD_r_@mSiO_2_ were calculated to be 5.2, 4.3, 3.8, 2.7, and 3.4%, respectively. The fluorescent intensities of the three types of CD_s_ decreased by <2.5% over 6 weeks, indicating that they were relatively stable. The reaction time was less than 10 min. Notably, CDs were all fabricated from modified pine needle sources using microwave assistance and can be termed in the green synthesis category with an environmentally friendly feature.

The particle sizes of iron oxide, iron oxide@mSiO_2_, CD_g_@mSiO_2_, and CD_r_@mSiO_2_, were 13, 45, 40, and 40nm. Figure 3c s depicts TEM images of iron oxide, iron oxide @mSiO_2_, and CD_g_@mSiO_2_. The fluorescent intensities of CD_b_ decrease with a marginal change of emission maximum as a modification with iron oxide @mSiO_2_ nanoparticles and antibodies (Figure 3b). The magnetic susceptibility of the magnetofluorescent nanoparticles was 27 emu g^−1^. The magnetic susceptibility value is much larger than those of literature (usually <10 emu g^−1^) and is more useful for separation application. Magnetofluorescent nanoparticles maintained good magnetic and fluorescent properties for analytical applications.

### 3.2. Magnetic Fluorescence-Linked Immunoassay

**Figure 4:**
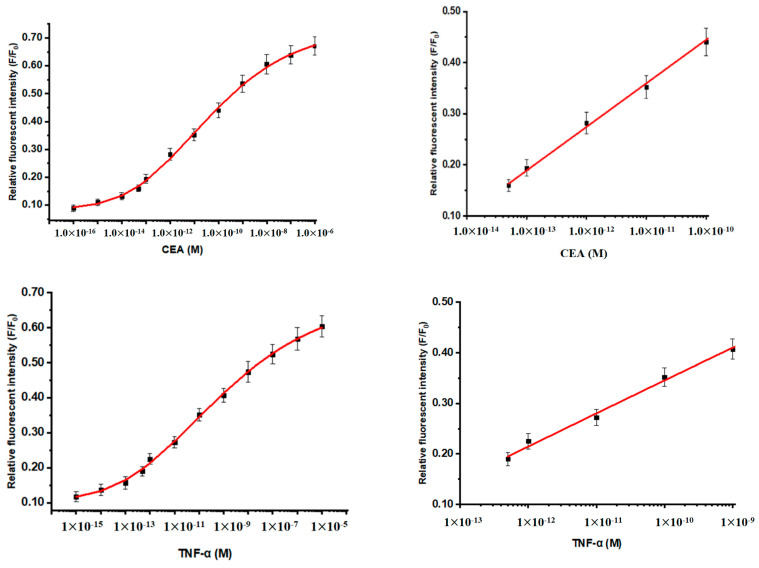
Reference plots of CEA and TNF-α for the magnetic fluorescence-linked immunoassay.

The magnetofluorescent nanoparticles exhibited efficient mixing, washing, and separation in the sandwich reaction. All nanoparticle reactions were highly effective under nearly homogeneous conditions. Figure 4 presents the reference plots of CEA and TNF-α for a magnetic fluorescence-linked immunoassay. CD_g_@mSiO_2_-Ab (475 nm) and CD_r_@mSiO_2_-Ab (660 nm) were used as Fo for the calculation of F/Fo. The formation of immunocomplex was used as F. The relative fluorescent intensity and concentrations of CEA and TNF-α exhibited the S shape characteristic of the sandwich immunoassay reaction. The linear range of CEA was 5.0 × 10^−13^ M (9 pg mL^−1^) to 1.0 × 10^−10^ M (18 ng mL^−1^) and the linear range of TNF-α was 5.0 × 10^−12^ M (8.5 pg mL^−1^) to 1.0 × 10^−9^ M (17 ng mL^−1^). The detection limits of CEA and TNF-α were 3.1 pg mL^−1^ and 2.8 pg mL^−1^, respectively. Table 1 presents a comparison of the detection limit and linear range obtained herein and in other studies [20,21,22,23,24,25,26]. The table contains one calorimetric method [25], two nanoparticle methods [20,23], and four electrochemical methods [21,22,24,26] from recent literature for comparison. The present method has a lower detection limit and wider linear range for CEA and TNF-α than do the compared approaches. The selectivity of the present method was tested by replacing CEA and TNF-α with 10^−10^ M of immunoglobulin G, hemoglobin, and ovalbumin in the sandwich reaction. The relative fluorescence intensities of these proteins were negligible (all <0.1, with no effect on the reference plots).

Table S1 presents a comparison of the proposed method with ELISA on the detection of CEA and TNF-α in spiked serum samples with three concentrations. The average (range) of the differences between the actual concentrations of the spiked samples and the concentrations detected using the proposed method was 5.6% (2.5–10%). The average (range) of the differences in the concentrations detected using the proposed method and ELISA was 7.9% (5.2–12%). These results indicate that both measurements from the proposed method and ELISA were within acceptable experimental error margins. Magnetic fluorescence-linked immunoassay has several advantages over those of ELISA. First, the antibody on the microplate has more available binding sites and a more efficient reaction with analytes without limitations of planar fixation. Second, magnetic nanoparticles can have faster separation and reconcentration under a magnetic field. Third, fluorescence detection has the potential for lower detection limits. The running time of the proposed method was approximately 25 min, one-third of the typical running time for ELISA. The high sensitivity of the proposed method originates from the efficient mixing of nanoparticles and its short running time of the sandwich reaction can be attributed to the magnetic concentrations used. The automation of this method is expectable for high throughput. The proposed method enables the rapid, sensitive, and cost-effective detection of biomarkers by using CD_s_ prepared from modified pine needles with ecologically friendly sources.

## 4. Conclusions

In this study, CD_s_ with blue, green, and red emissions were rapidly prepared from pine needles with some modifications. The prepared CDs can be employed in the rapid, simple, green, and economical detection of biomarkers for chemical analysis. Magnetic fluorescence-linked immunoassay with fluorescent CDs from modified pine needles was demonstrated for biomarker detection. The measurements of spiked biomarkers from the proposed method and ELISA were consistent and within acceptable experimental error margins. Compared with ELISA and other methods used in the literature, the magnetic fluorescence-linked immunoassay exhibited faster detection with a lower detection limit and/or wider linear range for CEA and TNF-α biomarkers. This method has the potential as an alternative method to ELISA for biomarker detections.

## Figures and Tables

**Table 1:** Comparison of detection limit and linear range for CEA and TNF-α.

Biomarker	Literature	Detection Limit (ng mL^−1^)	Linear Range (ng mL^−1^)	Ref.
CEA	Sens. Actuator. B chem. 2021, 342, 130075	0.013	0.05–40	[20]
	Anal. Chem. 2021, 93, 2916–2925	0.094	0.2–50	[21]
	ACS Apple Nano Mater. 2020, 3, 3449–3458	0.0054	0.03–6	[22]
	J Colloid Interface Sci. 2021, 601, 50–59	0.00352	0.05–40	[23]
	This study	0.0031	0.009–18	
TNF-α	Sens. Actuator. B chem. 2021, 336, 129747	0.005	0.005–0.20	[24]
	Bios. Bioelectron. 2019, 126, 82–87	0.014	0.014–2.0	[25]
	Sensor 2021, 20, 2857	0.078	0.1–100	[26]
	This study	0.0028	0.0085–17	

## Data Availability

Data can be available upon request from the authors.

## Supplementary Materials

The following supporting information can be downloaded at: https://www.mdpi.com/article/10.3390/nano12010066/s1. Table S1 Comparison of the Concentrations of CEA and TNF-α Detected Under the Proposed Method and the ELISA. Figure S1 The optimization of reaction temperature for a fixed time and reaction for a fixed temperature. Figure S2 TEM photographs of CD_b_, CD_g_, and CD_r_ nanoparticles. Figure S3. Infrared spectra of the three types of CDs.
