# Supplementary material for: Rapid Preparation of Fluorescent Carbon Dots from Pine Needles for Chemical Analysis
Source: Nanomaterials (Basel). 2021 Dec 28;12(1):66. doi: 10.3390/nano12010066 (PMC8746989; doi:10.3390/nano12010066)
Supplement: Supplementary file 1 [file nanomaterials-12-00066-s001.zip › nanomaterials-1504766-supplementary.pdf]

Supplementary Materials

Rapid Preparation of Fluorescent Carbon Dots from Pine Needles for Chemical Analysis

HweiYan Tsai [1,2,3], Kai Ying Chang [3], Wan Shing Lee [3] and C. Bor Fuh [3,*]

[1] Department of Medical Applied Chemistry, Chung Shan Medical University, Taichung 402, Taiwan; annetsai@csmu.edu.tw
[2] Department of Medical Education, Chung Shan Medical University Hospital, Taichung 402, Taiwan
[3] Department of Applied Chemistry, National Chi Nan University, Puli, Nantou 545, Taiwan; s108324501@mail1.ncnu.edu.tw (K.Y.C.); s108324902@mail1.ncnu.edu.tw (W.S.L.)
* Correspondence: cbfuh@ncnu.edu.tw; Tel.: +886-49-2919-779; Fax: 886-49-2917-956

Table S1. Comparison of the Concentrations of CEA and TNF-α Detected Under the Proposed Method and the ELISA (n = 5).

	Spiked	ELISA	This study
CEA (pg/mL)	400	385 ± 15	410 ± 13
	80	73 ± 4	77 ± 3
	16	17 ± 1	15 ± 1
TNF-α (pg/mL)	250	256 ± 12	243 ± 14
	50	52 ± 3	46 ± 2
	10	8 ± 1	9 ± 1

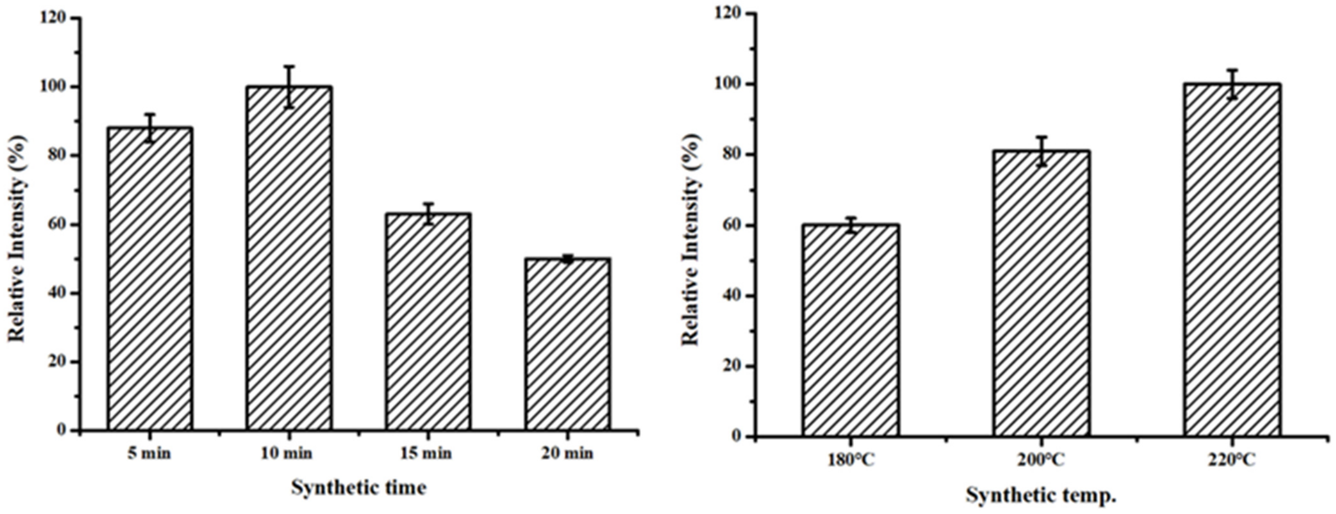

(**a**) (**b**)

Figure S1. The optimization of reaction temperature for a fixed time (**a**) and reaction time for a fixed temperature (**b**).

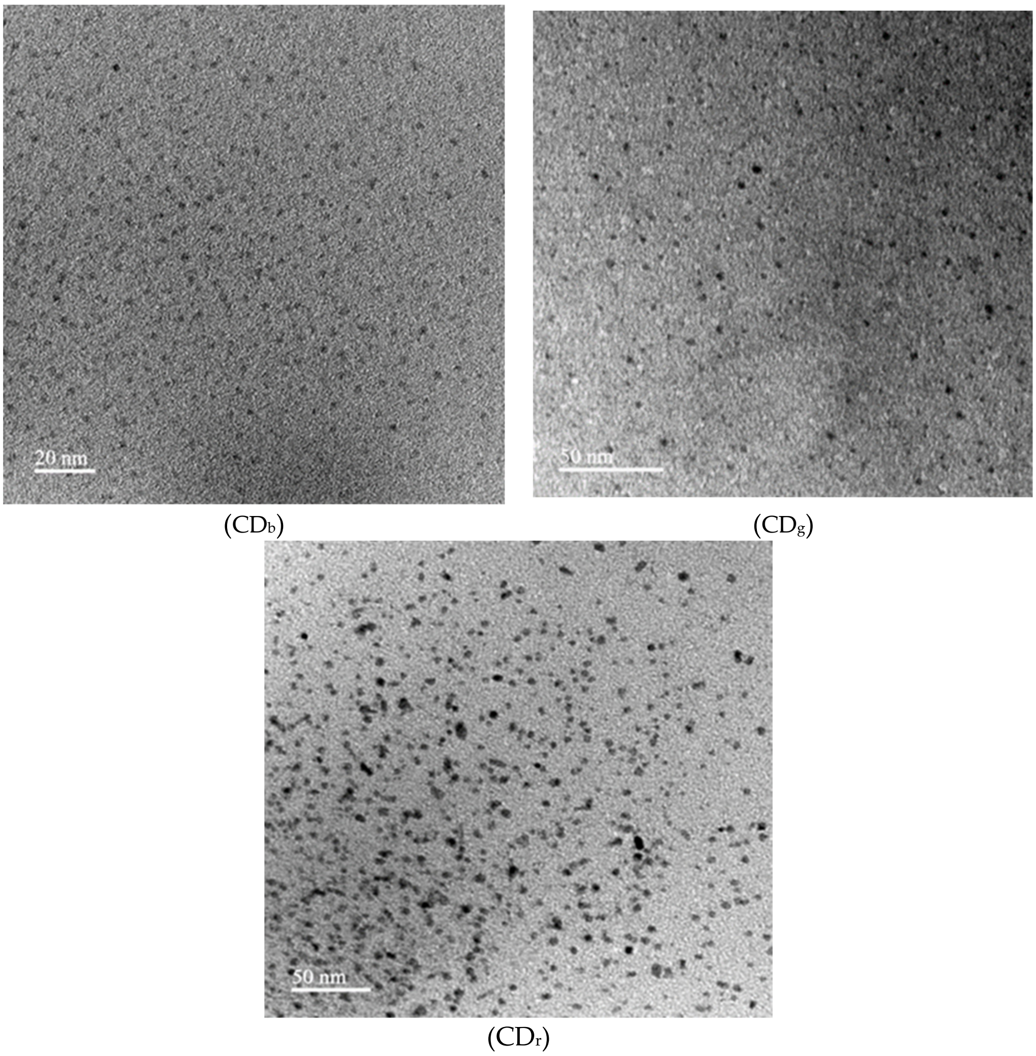

Figure S2. TEM photographs of CD$_b$, CD$_g$, and CD$_r$ nanoparticles.

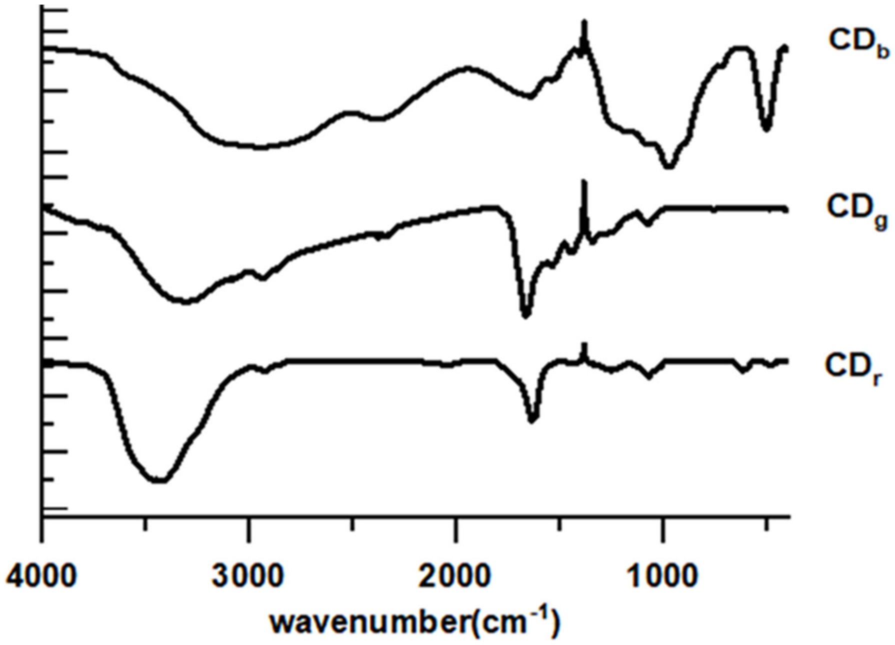

Figure S3. Infrared spectra of the three types of CDs.
